# Dimensions of Loneliness Among Older Adults in Osijek-Baranja County: Associations with Sociodemographic, Family, and Social Characteristics

**DOI:** 10.3390/healthcare14193165

**Published:** 2026-09-24

**Authors:** Monika Krpan Feher, Nikolina Farčić, Marin Mamić, Ivana Barać, Marija Barišić, Željko Mudri

**Affiliations:** 1 Faculty of Medicine, Josip Juraj Strossmayer University of Osijek, Josip Huttler Street 4, 31000 Osijek, Croatia; mfeher@mefos.hr; 2 Department of Nursing and Palliative Medicine, Faculty of Dental Medicine and Health Osijek, Josip Juraj Strossmayer University of Osijek, Car Hadrijan Street 10e, 31000 Osijek, Croatia; nfarcic@fdmz.hr (N.F.); mmamic@fdmz.hr (M.M.); ibarac@fdmz.hr (I.B.); mbarisic@fdmz.hr (M.B.); 3 Department of Neurosurgery, University Hospital Centre Osijek, Josip Huttler Street 4, 31000 Osijek, Croatia; 4 General County Hospital Požega, Osječka Street 107, 34000 Požega, Croatia

**Keywords:** aging, loneliness, older adults, social connection

## Abstract

**Highlights:**

**What are the main findings?**

- Poorer perceived quality of family contact was associated with higher social and family loneliness, even after adjustment for sociodemographic, family, and social characteristics.
- Men reported higher social loneliness than women in the bivariate analysis, although sex was not statistically significant in the adjusted model.

**What are the implications of the main findings?**

- The perceived quality of close relationships should be considered alongside the frequency of social contact when assessing loneliness in older adults.
- Brief questions about the perceived quality of family and social relationships could complement psychosocial assessments of older adults in community and public health nursing practice.

**Abstract:**

**Background/Objectives**: Loneliness among older adults is a public health concern associated with adverse health and well-being outcomes, yet evidence from eastern Croatia remains limited. This study examined social loneliness, family loneliness, and loneliness in romantic relationships among adults aged ≥65 years and their associations with sociodemographic, family, and social characteristics. **Methods**: This cross-sectional study included 431 community-dwelling older adults in Osijek-Baranja County from January to April 2025. Loneliness was assessed using the Croatian adaptation of the Social and Emotional Loneliness Scale. Group differences and correlations were examined using nonparametric methods, followed by separate multiple linear regression models with HC3 robust standard errors. **Results**: Median scores were 38 (29–47) for social loneliness, 20 (14–29) for family loneliness, and 41 (27–50) for loneliness in romantic relationships. Poorer perceived family-contact quality was associated with higher social and family loneliness in the adjusted models. Compared with good-quality contact, poor-quality contact was associated with social- and family-loneliness scores that were 19.66 and 25.52 points higher, respectively (both *p* < 0.001). Men and suburban residents had higher social loneliness in bivariate analyses, but sex and residence were not significant in the adjusted model. After adjustment for age, never-married, divorced, and widowed participants had romantic-loneliness scores 14.66, 21.55, and 15.58 points higher than married participants, respectively (all *p* < 0.001). Older age was weakly and positively correlated with social loneliness (ρ = 0.148; *p* = 0.002) and loneliness in romantic relationships (ρ = 0.194; *p* < 0.001). Family-contact frequency was not significant overall in the adjusted social and family loneliness models. **Conclusions**: Perceived family-contact quality and marital status were associated with distinct loneliness dimensions. Assessments should consider older adults’ experience of close relationships alongside contact frequency and structural indicators.

## 1. Introduction

Population ageing has turned loneliness among older adults into a pressing public health issue. When the WHO Commission on Social Connection released its landmark report in 2025, it estimated that around one in ten older people worldwide experience loneliness, while a further quarter are socially isolated, representing related but distinct conditions [1]. The prevalence of loneliness appears to be considerably higher in high-income countries, where Chawla et al. [2] found that approximately one in four older adults reported feeling lonely, although estimates vary substantially across European Union countries [3].

The consequences of loneliness extend well beyond emotional distress. Loneliness has been associated with a 26% higher risk of all-cause mortality [4], and, together with social isolation, with increased cardiovascular morbidity [5,6]. Loneliness has also been associated with cerebrovascular and Alzheimer’s disease pathology [7], and, together with social isolation, with sarcopenia [8]. Social isolation, which is closely related to loneliness, is listed by the WHO as a modifiable risk factor for dementia. More broadly, up to 45% of dementia cases may be attributable to potentially modifiable risk factors [9]. Therefore, loneliness is not merely an unpleasant subjective experience, as it is associated with measurable effects on physical and mental health in later life.

### 1.1. Factors Associated with Loneliness in Older Adults

Several factors have consistently been linked to loneliness among older adults. Partnership status is among the most important: a meta-analysis of the correlates of loneliness in late adulthood found that married individuals and those living with a partner reported lower levels of loneliness than those who were widowed, divorced, or had never married [10]. Living alone has also been associated with greater loneliness, although the quality of household relationships may be more important than the number of people living in the household [11,12]. Consistent with this, Norlin et al. found that models incorporating closeness, conflict, and perceived social support explained more variance in loneliness and showed a better fit than a model based on relationship quantity [13]. From a psychological perspective, these findings suggest that loneliness may reflect a discrepancy between the closeness and support individuals desire and what their existing relationships provide. Longitudinal findings by Kapelle and Monden further showed that loneliness increased in the year preceding widowhood or separation and peaked in the year of marital dissolution, with a larger increase around widowhood than separation [14]. Although loneliness gradually declined after widowhood, levels remained elevated among widowed men over the longer term [14]. Findings on sex differences in loneliness are mixed, with several studies reporting higher loneliness among women [15,16,17].

Participation in community organizations has likewise been associated with lower levels of loneliness. Shimoda et al. [18] and Nakahara and Yokoi [19] found that several factors, including community involvement, hobbies, and leisure activities, were associated with lower odds of loneliness. However, whether the frequency of engagement contributes independently of membership itself remains an open question [19].

Evidence on urban–rural differences in loneliness specifically is still limited, but a recent scoping review points out that older adults in rural areas face geographic and infrastructural barriers to social participation that likely shape their experience of loneliness in ways urban-based research has not captured [20].

### 1.2. Croatian Context and Study Rationale

Research addressing loneliness among older adults in Croatia remains limited. A cross-sectional study in northern Croatia examined social and emotional loneliness directly [17], while broader research from eastern Croatia has looked at successful ageing together with social connectedness [21] and subjective well-being [22], with loneliness appearing only as a single item within the wider ageing scale used in at least one of these studies [22], rather than as a primary outcome. Croatian studies conducted in specific institutional and clinical populations have also linked loneliness with lower perceived social support and lower life satisfaction [23,24]. Among nursing home residents during the COVID-19 pandemic, older adults without a partner reported greater loneliness, while greater perceived social support was associated with lower loneliness [23]. However, evidence concerning the distinct dimensions of loneliness among community-dwelling older adults in Croatia remains limited. Evidence specific to Osijek-Baranja County is lacking, although approximately one in four older adults in the County lives alone [25], a living arrangement consistently associated with an increased risk of loneliness.

Community and public health nurses are often in regular contact with older adults and may therefore be well positioned to identify those experiencing loneliness and facilitate appropriate assessment, support, or referral [26]. Understanding the prevalence and correlates of loneliness among community-dwelling older adults is therefore directly relevant to nursing practice, supporting the inclusion of a nursing perspective in research on loneliness at the community level.

### 1.3. Study Aims

The primary aim of this study was to examine social loneliness, family loneliness, and loneliness in romantic relationships among adults aged 65 years and older in Osijek-Baranja County and to assess their associations with participants’ sociodemographic, family, and social characteristics. Specifically, the study aimed to:

(1) examine the levels of social loneliness, family loneliness, and loneliness in romantic relationships;
(2) examine differences in these dimensions of loneliness according to selected sociodemographic, family, and social characteristics;
(3) assess the associations between age and each dimension of loneliness;
(4) among members of community organizations, examine the associations between the frequency of participation in organizational activities and each dimension of loneliness;
(5) assess the adjusted associations of the measured sociodemographic, family, and social characteristics with social loneliness, family loneliness, and loneliness in romantic relationships.

## 2. Materials and Methods

### 2.1. Study Design

This quantitative, cross-sectional study was conducted between January and April 2025 in Osijek-Baranja County, Croatia.

### 2.2. Participants

The study included 431 community-dwelling adults aged 65 years and older who resided in Osijek-Baranja County and were able to provide informed consent. In this study, community-dwelling adults were defined as individuals living in their own homes or in the homes of family members rather than in nursing homes or other residential care institutions. Individuals with diagnosed dementia, those residing in nursing homes, and those unable to provide informed consent were not eligible for inclusion. A previous diagnosis of dementia was identified based on information provided by the potential participant or a family member and, where available, supporting medical documentation. No formal cognitive screening instrument was administered. Other medical and psychiatric diagnoses were not systematically assessed and were not used as exclusion criteria. During the information and consent procedure, the principal investigator assessed whether potential participants appeared able to understand the study information, provide informed consent, and complete the questionnaire.

Participants were recruited between January and April 2025 using a non-probability convenience sampling method. Before recruitment, a target sample size of 382 participants was estimated assuming a population of 56,734, a 95% confidence level, a 5% margin of error, and a 50% response distribution [27]. Because a non-probability convenience sample was used, the above calculation was treated as a pragmatic recruitment target and not as evidence of population representativeness or sampling precision. Because multiple regression was a principal analytical approach, an additional minimum detectable effect analysis was performed using the available regression sample. For the most parameter-rich primary model (N = 396; 16 predictor parameters), with α = 0.05 and 80% power, the minimum detectable overall effect was Cohen’s f^2^ = 0.050, corresponding to a small effect (Supplementary Table S2).

During data screening, 22 respondents younger than 65 years, five respondents whose age was not reported and whose eligibility therefore could not be confirmed, one nursing home resident, and 32 respondents whose missing responses prevented the calculation of one or more loneliness subscale scores were excluded from the analysis. In total, 60 respondents were excluded, resulting in a final analytical sample of 431 participants. The median age was 72 years (Q1–Q3: 68–77), 272 (63.1%) participants were women, and 250 (58.0%) lived in urban areas.

### 2.3. Instruments

Data were collected in person using an anonymous questionnaire consisting of two components.

The first component was the Croatian version of the Social and Emotional Loneliness Scale adapted by Vera Ćubela Adorić and Marina Nekić. The instrument was obtained from the Collection of Psychological Scales and Questionnaires [28], and permission to use it was granted by Vera Ćubela Adorić.

The Social and Emotional Loneliness Scale comprises 36 items distributed across three subscales: social loneliness (14 items), family loneliness (11 items), and loneliness in romantic relationships (11 items). The term “loneliness” is not explicitly mentioned in the items, which include both positively and negatively worded statements. Examples of positively worded items are “I feel that I am part of my family” (family loneliness) and “There is someone who wants to share their life with me” (loneliness in romantic relationships); both items are reverse-scored. An example of a negatively worded item is “I do not have a friend who shares my views” (social loneliness). The subscale labels are not presented to respondents. Items assessing social loneliness are grouped together, whereas items assessing family loneliness and loneliness in romantic relationships are presented alternately, reflecting the conceptualization of the latter two domains as components of emotional loneliness. Respondents indicate their level of agreement with each statement on a seven-point Likert-type scale ranging from 1 (“strongly disagree”) to 7 (“strongly agree”). According to the scoring instructions, 23 items require reverse scoring so that higher scores consistently indicate greater loneliness. Each subscale score is calculated by summing the responses to its corresponding items, with higher scores indicating greater loneliness in that domain. Possible scores range from 14 to 98 for social loneliness and from 11 to 77 for both family loneliness and loneliness in romantic relationships. In the present study, ordinal McDonald’s omega coefficients were 0.919 for social loneliness, 0.917 for family loneliness, and 0.863 for loneliness in romantic relationships.

The second component comprised questions on sociodemographic, family, and social characteristics, including age, sex, marital status, household composition, place of residence, frequency and perceived quality of family contact, membership in a community organization, and, among members, the frequency of participation in organizational activities. Age was recorded in completed years, and sex was recorded as male or female. Marital-status categories were married, cohabiting, never married, divorced, and widowed, while household-composition categories were living alone, living with a spouse or partner, and living with other household members. Place of residence was self-reported as an urban area/city, a suburban settlement, or a rural area/village. Participants reported how often they saw their family, with four response options: daily, once or several times a week, once or several times a month, and once a year or less often. They also rated the perceived quality of family contact as good, partly good, or poor. Finally, participants reported whether they were members of a community organization and, if so, how many times per month they participated in its activities.

### 2.4. Data Collection Procedure

Before the data collection phase began, the research staff received targeted training in communication strategies and psychological approaches relevant to working with older adults. Data were collected in person in participants’ homes to provide privacy and a familiar and comfortable setting for questionnaire completion.

The principal investigator coordinated participant recruitment and data collection. Information about the study was disseminated through family members, primary care physicians, and representatives of local pensioners’ associations in Osijek-Baranja County, who were asked to share the information with older adults who might meet the eligibility criteria. In addition, researchers recruited potential participants by directly approaching potentially eligible older adults in neighboring households.

During participant recruitment, potential participants received written and verbal information about the purpose and procedures of the study, the voluntary nature of participation, the confidentiality of the collected data, and their right to withdraw at any time without consequences. Participants were given an opportunity to ask questions, and written informed consent was obtained before questionnaire administration.

Participants completed the anonymous questionnaire using the paper-and-pencil method. They first completed the self-administered Croatian version of the Social and Emotional Loneliness Scale. Sociodemographic, family, and social information was collected afterward to minimize the possibility that personal questions could influence responses to the loneliness scale [29]. During questionnaire completion, researchers remained available to clarify the instructions or the meaning of individual questions when necessary. Clarifications were provided in a neutral manner without suggesting or directing participants’ responses. No personally identifiable information was entered into the research database. Completion time was not limited. The average time required to complete the questionnaire was ten minutes per participant, ranging from eight to twelve minutes. The exact number of individuals who received information about the study, were assessed for eligibility, or declined participation was not systematically recorded. Therefore, a response proportion could not be calculated.

### 2.5. Ethical Considerations

The study was conducted in accordance with the ethical principles of the Declaration of Helsinki [30] and approved by the Committee for Ethical and Professional Affairs of Nurses and Nursing Technicians of University Hospital Centre Osijek (approval No. R1-96-7/2025; 3 January 2025). All potential participants received written information through a research notice form and were also informed verbally about the purpose of the study, the data collection procedures, the voluntary nature of participation, the confidentiality of the collected data, and their right to withdraw from the study at any time without providing a reason or experiencing any consequences.

Participants were given an opportunity to ask questions and clarify any uncertainties before deciding whether to participate. Written informed consent was obtained from all participants before questionnaire administration. To protect participants’ anonymity, the questionnaires did not contain personally identifying information and were stored separately from the signed informed consent forms.

### 2.6. Data Analysis

Descriptive statistical methods were used to summarize the distribution of the study variables. Categorical variables were presented as absolute and relative frequencies. Participants’ age and, among members of community organizations, the frequency of participation in organizational activities were presented as the median and interquartile range, Mdn (Q1–Q3). Scores on the social loneliness, family loneliness, and loneliness in romantic relationships subscales were presented as the mean and standard deviation and as the median and interquartile range.

The internal consistency of the subscales was assessed using ordinal McDonald’s omega coefficients calculated from polychoric correlations. Normality was evaluated using the Shapiro–Wilk test. Because the scores on all three subscales deviated significantly from a normal distribution, nonparametric tests were used in the bivariate inferential analyses.

Differences in loneliness subscale scores between two independent groups were examined using the Mann–Whitney U test, whereas differences among three or more groups were examined using the Kruskal–Wallis test. Statistically significant Kruskal–Wallis tests were followed by Dunn’s post hoc pairwise comparisons with Holm adjustment for multiple testing. Effect sizes were expressed as the rank-biserial correlation coefficient (r_rb_) for Mann–Whitney U tests, epsilon-squared (ε^2^) for overall Kruskal–Wallis tests, and the rank-biserial correlation coefficient for the corresponding pairwise group contrasts.

Associations between age and the three loneliness dimensions were examined using Spearman’s rank correlation coefficient. Among participants who were members of community organizations, Spearman’s rank correlation coefficient was also used to assess the association between the frequency of participation in organizational activities and each loneliness dimension.

Age was analysed as a continuous variable. Sex and community-organization membership were treated as binary nominal variables. Place of residence, marital status, household composition, family-contact frequency, and perceived family-contact quality were treated as categorical variables. Although family-contact frequency was collected in ordered categories, it was entered into the regression models as an unordered categorical factor rather than as a continuous score. Household composition was collapsed into three categories: living alone, living with a spouse/partner, and living with other household members. Categorical predictors were indicator (dummy) coded. The reference categories were male sex, urban residence, married status, living with a spouse/partner, daily family contact, good perceived family-contact quality, and non-membership in a community organization, as applicable to each model. Place of residence was self-reported as urban, suburban, or rural; no objective population-density measure was used. Frequency of participation in organizational activities was treated as a count variable representing days per month and was analysed only among community-organization members; non-members were treated as not applicable (NA) rather than assigned a value of zero.

The social loneliness model included age, sex, residence, marital status, household composition, family-contact frequency and quality, and community-organization membership. The family loneliness model included age, marital status, household composition, and family-contact frequency and quality, whereas the romantic loneliness model included age and marital status. Overall effects of categorical predictors were assessed using robust Wald F-tests.

Predictor sets were defined according to the conceptual domain of each loneliness dimension and the study aims rather than selected according to bivariate statistical significance. The social-loneliness model therefore included the broader set of measured sociodemographic, family, and social characteristics; the family-loneliness model focused on age and family/household characteristics; and the romantic-loneliness model focused on age and marital status as the measured characteristics most directly related to that domain.

Regression assumptions and model diagnostics, including linearity, residual distribution, homoscedasticity, multicollinearity, model specification, and influential observations, were examined before interpretation. Because heteroscedasticity was identified in some models, HC3 robust standard errors were applied in all regression analyses. Results are presented as unstandardized regression coefficients (B), HC3 robust standard errors, 95% confidence intervals, and *p*-values. Model performance was assessed using the adjusted coefficient of determination (adjusted R^2^). Missing data were not imputed. Statistical significance was set at *p* < 0.05.

Additional sensitivity analyses were conducted to assess robustness. All three outcomes were re-estimated using the same measured covariate set (age, sex, residence, marital status, household composition, family-contact frequency and quality, and community-organization membership). Models involving marital status were also repeated after excluding the small cohabiting (n = 8) and never-married (n = 12) categories. Because only 16 participants rated family-contact quality as poor, the social and family loneliness models were repeated after excluding this category and after combining partly good and poor responses into a single ‘not fully good’ category. To assess dependence of the romantic-loneliness model on marital status, additional models were estimated without marital status. Finally, primary models were refitted after excluding observations with Cook’s distance > 4/N. All sensitivity models used HC3 robust standard errors and robust Wald F-tests, and they were treated as supplementary robustness checks rather than replacements for the primary models. Missing data were handled by complete-case analysis within each model; no imputation was performed. Detailed results are provided in Supplementary Tables S1–S9. Interpretation of these sensitivity analyses focused on the direction and magnitude of the estimated coefficients and their 95% confidence intervals rather than on omnibus model significance alone. Estimates involving the small cohabiting (n = 8), never-married (n = 12), and poor family-contact quality (n = 16) categories were regarded as exploratory.

Statistical analyses were performed using R, version 4.6.1 (R Foundation for Statistical Computing, Vienna, Austria), through RStudio Desktop, version 2026.07.1 (Posit Software, PBC, Boston, MA, USA). The following R packages were used: haven, dplyr, tidyr, tibble, purrr, stringr, psych, FSA, car, lmtest, sandwich, MASS, and writexl. The pwr package was additionally used for the regression sample-size sensitivity analysis.

## 3. Results

Detailed participant characteristics are presented in Table 1. Among members of community organizations, the median frequency of participation in organizational activities was 3 (Q1–Q3: 2–5).

The median scores were 38 (Q1–Q3: 29–47) for social loneliness, 20 (Q1–Q3: 14–29) for family loneliness, and 41 (Q1–Q3: 27–50) for loneliness in romantic relationships. Scores on all three subscales deviated significantly from a normal distribution (Table 2).

Social loneliness was higher among men than women and differed by place of residence, with the Holm-adjusted post hoc comparison indicating higher social loneliness among participants from suburban than urban areas.

Perceived family-contact quality also differed across groups: participants reporting partly good or poor contact had higher social loneliness than those reporting good contact, and the poor-contact group also had higher social loneliness than the partly good group. Effect sizes were small for sex and place of residence and moderate for perceived family-contact quality (Table 3).

Family loneliness did not differ significantly according to marital status or household composition. It differed according to family-contact frequency and perceived family-contact quality. Holm-adjusted pairwise comparisons showed higher family loneliness among participants with family contact once a year or less often than among those with daily or weekly contact. Participants reporting partly good or poor family contact also had higher family loneliness than those reporting good contact, and the poor-contact group had higher family loneliness than the partly good group. The overall effect was small for contact frequency and large for perceived contact quality (Table 4).

Loneliness in romantic relationships differed according to marital status. Holm-adjusted pairwise comparisons showed lower loneliness among married participants than among never-married, divorced, and widowed participants, and lower loneliness among cohabiting participants than among never-married, divorced, and widowed participants. The overall effect size was large (Table 5).

A weak positive association was observed between age and social loneliness (ρ = 0.148; *p* = 0.002) and between age and loneliness in romantic relationships (ρ = 0.194; *p* < 0.001) (Table 6).

Missingness was low at the variable level. Missing data comprised 10 marital-status values (2.3%), 8 household-composition values (1.9%), 17 family-contact-frequency values (3.9%), 7 family-contact-quality values (1.6%), and 2 romantic-loneliness scores (0.5%); other variables entering the primary models were complete. Complete-case analysis yielded N = 396 for the social and family loneliness models and N = 419 for the romantic-loneliness model (Supplementary Table S1).

The social loneliness model was statistically significant (FHC3(16, 379) = 4.49; *p* < 0.001) and explained 13.0% of the variance in social loneliness (adjusted R^2^ = 0.130). Older age was associated with significantly higher social loneliness (B = 0.37; *p* = 0.014). Marital status was statistically significant as an overall predictor of social loneliness (*p* < 0.001). Compared with married participants as the reference category, cohabiting participants had significantly lower social loneliness (B = −9.83; *p* < 0.001), as did never-married participants (B = −9.63; *p* = 0.008), after adjustment for all other predictors included in the model.

Perceived quality of family contact was statistically significant as an overall predictor of social loneliness (*p* < 0.001). Compared with participants who rated their family contact as good, those who rated it as partly good (B = 7.38; *p* < 0.001) or poor (B = 19.66; *p* < 0.001) had higher social loneliness after adjustment for the other variables included in the model. Family-contact frequency was not statistically significant as an overall predictor (FHC3(3, 379) = 1.83; *p* = 0.142). Although the individual contrast between contact once a year or less often and daily contact yielded *p* = 0.049, this contrast tests a single comparison, whereas the robust Wald F-test evaluates the categorical predictor jointly. Because the overall test was not statistically significant, the isolated contrast was not interpreted as evidence of an overall association and should be interpreted cautiously (Table 7).

The family loneliness model was statistically significant (FHC3(12, 383) = 10.65; *p* < 0.001) and explained 27.0% of the variance in family loneliness (adjusted R^2^ = 0.270). Perceived quality of family contact was the only statistically significant overall predictor of family loneliness (FHC3(2, 383) = 44.18; *p* < 0.001). Compared with participants who rated their family contact as good, those who rated it as partly good (B = 7.17; *p* < 0.001) or poor (B = 25.52; *p* < 0.001) had higher family loneliness after adjustment for the other variables included in the model. Although never-married participants had significantly lower family loneliness than married participants (B = −6.82; *p* = 0.018), marital status was not statistically significant as an overall predictor (FHC3(4, 383) = 1.45; *p* = 0.216) (Table 8).

The loneliness in romantic relationships model was statistically significant (FHC3(5, 413) = 54.73; *p* < 0.001) and explained 38.5% of the variance in loneliness in romantic relationships (adjusted R^2^ = 0.385). Older age was associated with significantly higher loneliness in romantic relationships (B = 0.24; *p* = 0.005). Marital status was statistically significant as an overall predictor of loneliness in romantic relationships (FHC3(4, 413) = 53.50; *p* < 0.001). Compared with married participants, never-married participants (B = 14.66; *p* < 0.001), divorced participants (B = 21.55; *p* < 0.001), and widowed participants (B = 15.58; *p* < 0.001) had higher loneliness in romantic relationships after adjustment for age (Table 9).

### Sensitivity Analyses

Sensitivity analyses were evaluated primarily on the direction, magnitude, and 95% confidence intervals of the estimated coefficients rather than on omnibus model significance alone. When the same measured covariate set was applied to all three outcomes, the main coefficient patterns were broadly consistent with the primary analyses. For social loneliness, partly good and poor family contact remained associated with higher scores (B = 7.38, 95% CI [3.40; 11.36] and B = 19.66, 95% CI [9.53; 29.79], respectively). For family loneliness, the corresponding estimates were B = 7.05, 95% CI [4.30; 9.81], and B = 23.97, 95% CI [17.16; 30.77]. In the romantic-loneliness model using the same covariate set, the estimates for divorced and widowed participants remained positive (B = 16.21, 95% CI [9.19; 23.23] and B = 9.88, 95% CI [5.52; 14.23]), whereas the estimate for never-married participants was more uncertain (B = 8.12, 95% CI [−0.10; 16.34]) (Supplementary Table S3).

After excluding the cohabiting and never-married categories, the estimates for divorced and widowed participants in the romantic-loneliness model were essentially unchanged (B = 21.54, 95% CI [16.48; 26.60] and B = 15.60, 95% CI [13.26; 17.93], respectively). The family-contact quality findings were also stable when the 16 participants reporting poor contact were excluded: partly good versus good contact remained associated with higher social loneliness (B = 7.22, 95% CI [3.24; 11.20]) and family loneliness (B = 7.05, 95% CI [4.27; 9.84]). When partly good and poor contact were combined, not-fully-good contact was associated with higher social loneliness (B = 9.31, 95% CI [5.33; 13.28]) and family loneliness (B = 10.27, 95% CI [7.21; 13.32]) (Supplementary Tables S4–S6).

Removing marital status from the romantic-loneliness model reduced the adjusted R^2^ from 0.385 in the primary model to 0.358 when the remaining measured covariates were included, while substantial variation remained associated with other measured characteristics. This robustness analysis does not remove the conceptual overlap between partnership status and romantic loneliness and was therefore interpreted cautiously. Excluding observations with Cook’s distance > 4/N did not materially alter the overall pattern of the primary findings (Supplementary Tables S7–S9). Because the cohabiting (n = 8), never-married (n = 12), and poor-contact (n = 16) groups were small, estimates involving these categories are considered exploratory and should be interpreted with particular attention to confidence-interval width rather than *p*-values alone.

## 4. Discussion

This study examined social, family, and romantic loneliness among community-dwelling adults aged 65 years and older in Osijek-Baranja County, Croatia. Among the variables included in the adjusted models, perceived family-contact quality was associated with both social and family loneliness, whereas marital status showed the clearest pattern of group differences in loneliness in romantic relationships.

In the bivariate analysis, men reported higher social loneliness than women, although the effect size was small. However, sex was not independently associated with social loneliness after adjustment for the other variables included in the regression model. This finding contrasts with much of the existing literature. Neuberg et al. [17], using the same instrument in a sample of older adults from northern Croatia, found that women reported higher levels of loneliness. Pagan and Malo [15] similarly reported higher loneliness among older women in a European sample, and Ratcliffe et al. [31], drawing on data from the English Longitudinal Study of Ageing, found that women scored higher on the UCLA Loneliness Scale, although men were less likely to directly acknowledge feeling lonely. One possible explanation for this discrepancy lies in the dimension-specific nature of the present finding: the observed sex difference concerned social loneliness, which captures the adequacy of friendships and broader social networks. Ratcliffe et al. [31] reported that older men had poorer-quality friendships and fewer close relationships outside of their partnership, which may make them particularly vulnerable to social loneliness even when overall loneliness scores appear lower on global measures. Cultural expectations around masculinity and reluctance to disclose emotional distress may further suppress self-reported loneliness on direct questions while leaving dimension-specific scores unaffected [31]. Pagan and Malo [15] also noted that sex differences in loneliness vary with age and measurement approach.

In a Turkish sample of 610 older adults, Gümüş Demir and Yılmaz [32] found that participants living in villages or towns had higher loneliness and depression scores than urban residents. In the present study, participants from suburban areas reported higher social loneliness than those from urban areas, whereas no significant difference was found between rural and urban participants. This finding should be interpreted cautiously because the suburban subgroup was relatively small (n = 61), and the study did not measure local availability of services, transport, mobility, neighborhood cohesion, or opportunities for social participation. The observed difference may reflect characteristics of the sample or unmeasured contextual factors rather than a general suburban pattern. Further studies using larger samples and direct measures of neighborhood and community resources are needed. Older adults in suburban areas may have more limited mobility and fewer opportunities for regular social participation, which could contribute to higher social loneliness. Irwin et al. [20] noted in their scoping review that older adults in rural areas face geographic and infrastructural barriers including limited transport, workforce shortages, and scarce community-based programs that can hinder social participation, suggesting that the rural context shapes social isolation in ways that warrant greater research attention.

Perceived family-contact quality was associated with both social and family loneliness in the respective adjusted models. Compared with participants who rated their family contact as good, those who rated it as poor had estimated social-loneliness scores that were 19.66 points higher and family-loneliness scores that were 25.52 points higher. This pattern is consistent with the findings of Norlin et al. [13], who studied 474 adults aged 77 years and older in Sweden and reported that relationship quality and quantity were both associated with loneliness. Models incorporating relationship quality, assessed through closeness, conflict, and perceived social support, explained more variance and showed better fit than the model based on relationship quantity. Their findings support the relevance of relationship quality, although differences in study design, measurement, and population limit direct comparison with the present study. Norlin et al. [13] also reported associations between partnership status, relationship conflict, relationships with children, and loneliness.

Wilson et al. [33] similarly found that perceived social support was associated with loneliness across two independent samples, whereas social network size and received emotional or instrumental support were not consistently associated with loneliness. Contact frequency was associated with loneliness in both samples, while network closeness was associated with loneliness in one sample. In the present study, family-contact frequency was associated with family loneliness in the bivariate analysis but not in the adjusted model, whereas perceived family-contact quality remained significantly associated. From this perspective, frequent family contact may coexist with loneliness when the relationship does not provide the closeness or support desired by the individual [13].

Family loneliness was not significantly associated with marital status or household composition in the bivariate analyses, and these variables did not show significant overall associations in the adjusted model. This finding is consistent with Neuberg et al. [17], who similarly reported that marital status was not associated with family loneliness. By contrast, perceived family-contact quality was associated with family loneliness and showed a relatively large bivariate effect size. Perceived family-contact quality and family loneliness both reflect participants’ subjective experiences of their family relationships. Because perceived family-contact quality was assessed using a single subjective item and loneliness was also measured by self-report, conceptual overlap and shared method variance may partly explain the strength of the observed association. Therefore, the finding should be interpreted as an association between related subjective experiences rather than as evidence that perceived contact quality independently determines family loneliness. Furthermore, the coefficients for poor versus good family-contact quality were based on only 16 participants in the poor-contact category and should not be interpreted as precise estimates of the magnitude of the association. However, its association with family loneliness remained statistically significant in sensitivity analyses that excluded this category or combined the poor and partly good categories, supporting the robustness of the overall association.

Loneliness in romantic relationships showed a clear association with marital status, and the adjusted model explained 38.5% of the variance in this outcome. Compared with married participants, divorced and widowed participants had higher loneliness scores in romantic relationships, while the estimate for cohabiting participants did not differ significantly from that for married participants in the adjusted model. However, the loneliness-in-romantic-relationships subscale directly assesses the presence and perceived adequacy of a romantic relationship. Therefore, its strong association with marital status and the proportion of variance explained may partly reflect conceptual overlap between the predictor and outcome. The observed differences should consequently be interpreted descriptively rather than as evidence of an independent causal effect of marital status. These group differences are broadly consistent with previous studies reporting higher loneliness among widowed and divorced older adults. Liu et al. [34], analyzing data from the National Health and Aging Trends Study, found that divorced and widowed older adults reported higher loneliness than their married counterparts during the COVID-19 pandemic, and that these differences were not explained by changes in social participation or contact frequency. Vedder et al. [35], in a systematic review of 38 studies, confirmed that widowhood was associated with greater loneliness than other marital statuses, with the widowed being lonelier on average than divorced individuals. Kapelle and Monden [14] further demonstrated that loneliness increased in the year before widowhood or separation and that widowed men were more strongly affected than widowed women, although sex differences were negligible for separation. Because of its cross-sectional design, the present study could not examine the temporal dynamics that Kapelle and Monden [14] identified as central to understanding how loneliness evolves over marital transitions.

Older age showed weak positive cross-sectional associations with social loneliness and loneliness in romantic relationships, but not with family loneliness. Given the small correlation coefficients, these associations should be interpreted as modest in magnitude. Neuberg et al. [17] found that participants aged 85 years and older had higher scores for loneliness in romantic relationships than participants aged 65–84 years, whereas no statistically significant age-group differences were observed for social or family loneliness. The present study used age as a continuous variable and found weak positive associations with social loneliness and loneliness in romantic relationships. These findings are not directly comparable because the two studies operationalized age differently. The divergence in the social loneliness–age association between the two studies may reflect differences in sample composition: Neuberg et al. [17] included both institutionalized and community-dwelling older adults, whereas the present study was limited to community-dwelling individuals. Di Gessa et al. [16], using a 10-year English panel, identified heterogeneous loneliness trajectories in later life, suggesting that age-related increases in loneliness are not universal but depend on individual and contextual factors that accumulate over time.

Community-organization membership was not associated with social loneliness, and participation frequency among organization members was not associated with any of the three loneliness dimensions. These findings differ from studies that used broader or more detailed measures of social participation. Chu et al. [36], in a Chinese sample of 629 older adults, found that social participation was significantly and negatively correlated with loneliness after adjusting for demographic and health variables. Shimoda et al. [18] identified distinct patterns of social participation that were differentially associated with loneliness in Japanese older adults. However, Nakahara and Yokoi [19] observed that while meaningful social participation was associated with lower loneliness and reduced cognitive decline, the frequency of engagement did not independently predict these outcomes, raising the question of whether it is the act of participation itself, its subjective meaning, or the social connections it fosters that matters most. The present study measured frequency of participation rather than its perceived meaningfulness, which may explain the null finding. It is also possible that the types of community organizations available in Osijek-Baranja County, and the roles available to older adults within them, do not provide the depth of social connection needed to influence loneliness, or that self-selection into organizational membership is itself driven by factors associated with loneliness in ways that obscure a protective effect.

One finding requiring caution is the lower adjusted social-loneliness score observed among cohabiting and never-married participants compared with married participants. These estimates were based on very small subgroups, eight cohabiting and twelve never-married participants, and should therefore be regarded as imprecise and hypothesis-generating. Findings involving cohabiting and never-married participants should be considered exploratory. The direction of these adjusted associations was not consistent with the corresponding descriptive pattern and may reflect sparse categories, sampling variability, residual confounding, or model instability. Because the study was not designed to investigate these subgroup differences, no substantive interpretation is offered. Larger studies with sufficient numbers in each marital-status category are needed to determine whether these associations are reproducible.

This study has several strengths, including the use of a multidimensional loneliness instrument distinguishing between social, family, and romantic loneliness, the inclusion of effect-size estimates, and the use of heteroscedasticity-consistent standard errors. The study also has limitations. Its cross-sectional design precludes causal inference, and the non-probability convenience sampling method limits the generalizability of the findings. Because the numbers of individuals approached, assessed for eligibility, and declining participation were not systematically recorded, a response proportion could not be calculated and the extent of potential selection bias could not be determined. Data were self-reported, and reporting tendencies may have influenced responses. Previous research has shown that older men may be less likely than older women to disclose loneliness in response to direct questions [31]. Because the present study did not assess social desirability or reluctance to disclose loneliness, the extent of such bias in the current sample is unknown. The study was conducted in a single county in eastern Croatia, and the findings may not be generalizable to other regions or countries. Cognitive function was not formally assessed, and although participants with diagnosed dementia were excluded, undiagnosed cognitive impairment may have affected questionnaire responses.

Several potentially relevant factors, including depression, physical health, recent bereavement, mobility, and social network size, were not assessed. Therefore, the adjusted associations are limited to the variables included in the models, and residual confounding cannot be excluded. In addition, the small number of participants in some exposure categories may have produced unstable estimates and limited the reliability of subgroup-specific comparisons. Although the sensitivity analyses supported the overall patterns of findings, estimates involving these categories should be considered exploratory. Finally, given the number of comparisons performed, individual *p*-values, particularly those close to the conventional 0.05 threshold, should be interpreted cautiously and considered alongside effect sizes, confidence intervals, and the overall pattern of findings.

Future research should employ longitudinal designs to disentangle the temporal relationships between social contact quality, marital transitions, and loneliness trajectories. Intervention studies are needed to test whether strategies focused on improving the quality and emotional depth of family and social relationships are more effective than those that simply increase the frequency of contact or the number of social activities. Qualitative research could provide valuable insight into how older adults themselves define and experience relationship quality, and how community nursing services might be structured to address loneliness in ways that are acceptable and meaningful to this population. Larger national samples using probability-based sampling and including both urban and rural areas would help clarify the geographic patterns identified in this study and the present null finding regarding community participation.

## 5. Conclusions

This cross-sectional study examined three dimensions of loneliness among community-dwelling older adults in Osijek-Baranja County, Croatia. Perceived family-contact quality was associated with social and family loneliness in the adjusted models, while marital status showed the clearest pattern of group differences in loneliness in romantic relationships. Family-contact frequency, community-organization membership, and participation frequency did not show consistent adjusted associations with the loneliness dimensions examined. These findings suggest that perceived relationship quality should be considered alongside structural and frequency-based indicators of social connection, but they do not establish causality or relative importance among these indicators. Future longitudinal and intervention studies should determine whether changes in relationship quality or social contact are followed by changes in loneliness and whether relationship-focused interventions are effective.

## Figures and Tables

**Table 1:** Sociodemographic, Family, and Social Characteristics of Participants.

Characteristic	Category	n (%)/Mdn (Q1–Q3)
Sex	Male	159 (36.9)
	Female	272 (63.1)
Place of residence	Urban area	250 (58.0)
	Suburban area	61 (14.2)
	Rural area	120 (27.8)
Marital status (n = 421)	Married	208 (49.4)
	Cohabiting	8 (1.9)
	Never married	12 (2.9)
	Divorced	34 (8.1)
	Widowed	159 (37.8)
Household composition (n = 423)	Living alone	143 (33.8)
	Living with a spouse/partner	144 (34.0)
	Living with other household members	136 (32.2)
Frequency of family contact (n = 414)	Daily	129 (31.2)
	Once or several times a week	156 (37.7)
	Once or several times a month	69 (16.7)
	Once a year or less often	60 (14.5)
Perceived quality of family contact (n = 424)	Good	334 (78.8)
	Partly good	74 (17.5)
	Poor	16 (3.8)
Membership in a community organization	Yes	149 (34.6)
	No	282 (65.4)
Chronological age	Years	72 (68–77)
Frequency of participation in organizational activities (n = 149)	Days per month	3 (2–5)

**Table 2:** Descriptive Statistics and Distribution Normality.

Subscale	M (SD)	Mdn (Q1–Q3)	Min–Max	Shapiro–Wilk W
Social loneliness (n = 431)	39.15 (14.53)	38 (29–47)	14–92	0.960 *
Family loneliness (n = 431)	22.39 (10.68)	20 (14–29)	11–65	0.887 *
Loneliness in romantic relationships (n = 429)	38.58 (14.29)	41 (27–50)	11–75	0.972 *

* *p* < 0.001.

**Table 3:** Differences in social loneliness according to sociodemographic, family, and social characteristics.

Characteristic	Category	n	Mdn (Q1–Q3)	U/H (df)	*p*	r_rb_/ε^2^
Sex	Male	159	40 (31–51)	24,545	0.019	0.135
	Female	272	36.5 (27.5–45.5)			
Place of residence	Urban area	250	37 (28–46)	6.59 (2)	0.037	0.011
	Suburban area	61	43 (34–51)			
	Rural area	120	37 (29–44.5)			
Marital status	Married	208	38 (29–46.5)	6.26 (4)	0.180	0.005
	Cohabiting	8	25.5 (22.5–34.5)			
	Never married	12	35 (28–41)			
	Divorced	34	38.5 (28–52)			
	Widowed	159	37 (30–46)			
Household composition	Living alone	143	38 (30–47)	0.19 (2)	0.907	0.000
	Living with a spouse/partner	144	38 (27.5–46)			
	Living with other household members	136	36 (28–48.5)			
Frequency of family contact	Daily	129	38 (29–49)	3.39 (3)	0.335	0.001
	Once or several times a week	156	38 (29.5–47)			
	Once or several times a month	69	39 (29–48)			
	Once a year or less often	60	34.5 (26.5–42.5)			
Perceived quality of family contact	Good	334	36 (28–45)	28.23 (2)	<0.001	0.062
	Partly good	74	41.5 (32–54)			
	Poor	16	51 (43–55)			
Membership in a community organization	No	282	38 (29–50)	23,044.50	0.098	0.097
	Yes	149	36 (28–44)			

Note: U—Mann–Whitney U statistic; H—Kruskal–Wallis statistic.

**Table 4:** Differences in family loneliness according to sociodemographic, family, and social characteristics.

Characteristic	Category	n	Mdn (Q1–Q3)	U/H (df)	*p*	r_rb_/ε^2^
Marital status	Married	208	19 (14–26)	4.63 (4)	0.327	0.002
	Cohabiting	8	20.5 (17–30)			
	Never married	12	21.5 (13–31.5)			
	Divorced	34	22 (14–38)			
	Widowed	159	20 (14–28)			
Household composition	Living alone	143	22 (16–31)	5.45 (2)	0.066	0.008
	Living with a spouse/partner	144	19 (13.5–26.5)			
	Living with other household members	136	19.5 (13.5–25)			
Frequency of family contact	Daily	129	18 (13–28)	14.36 (3)	0.002	0.028
	Once or several times a week	156	19 (12–24)			
	Once or several times a month	69	23 (16–31)			
	Once a year or less often	60	23 (17–36.5)			
Perceived quality of family contact	Good	334	18 (12–24)	69.69 (2)	<0.001	0.161
	Partly good	74	24.5 (19–35)			
	Poor	16	43 (36.5–53)			

Note: U—Mann–Whitney U statistic; H—Kruskal–Wallis statistic.

**Table 5:** Differences in loneliness in romantic relationships according to marital status.

Characteristic	Category	n	Mdn (Q1–Q3)	H (df)	*p*	r_rb_/ε^2^
Marital status	Married	207	28 (20–39)	165.40 (4)	<0.001	0.390
	Cohabiting	8	22.5 (16–37)			
	Never married	12	48 (39.5–52.5)			
	Divorced	34	53 (46–58)			
	Widowed	158	47 (41–53)			

Note: H—Kruskal–Wallis statistic.

**Table 6:** Associations of age and frequency of participation in community organization activities with the loneliness subscale scores.

Sample	Variable	Statistic	Social Loneliness	Family Loneliness	Loneliness in Romantic Relationships
Full sample	Age	ρ	0.148	0.041	0.194
		*p*	0.002	0.390	<0.001
		n	431	431	429
Members of community organizations	Frequency of participation in organization activities	ρ	−0.014	0.027	−0.016
		*p*	0.862	0.745	0.842
		n	149	149	148

Note: ρ—Spearman’s rank correlation coefficient; n—number of participants.

**Table 7:** Multiple linear regression model of social loneliness.

Predictor	B	SEHC3	95% CI	FHC3 (df)	*p*
Age	0.37	0.15	[0.07; 0.66]	6.05 (1, 379)	0.014
Sex–overall predictor test	–	–	–	1.73 (1, 379)	0.190
Female vs. male	−2.03	1.54	[−5.07; 1.01]	–	0.190
Place of residence–overall predictor test	–	–	–	0.33 (2, 379)	0.719
Suburban vs. urban area	1.62	2.13	[−2.57; 5.81]	–	0.447
Rural vs. urban area	−0.16	1.85	[−3.79; 3.47]	–	0.931
Marital status–overall predictor test	–	–	–	5.73 (4, 379)	<0.001
Cohabiting vs. married	−9.83	2.91	[−15.56; −4.10]	–	<0.001
Never married vs. married	−9.63	3.64	[−16.78; −2.48]	–	0.008
Divorced vs. married	3.00	4.06	[−4.99; 10.99]	–	0.461
Widowed vs. married	−0.03	2.51	[−4.97; 4.91]	–	0.990
Household composition–overall predictor test	–	–	–	0.06 (2, 379)	0.944
Living alone vs. living with a spouse/partner	−0.96	2.90	[−6.67; 4.75]	–	0.741
Living with other household members vs. living with a spouse/partner	−0.52	2.16	[−4.76; 3.72]	–	0.809
Frequency of family contact–overall predictor test	–	–	–	1.83 (3, 379)	0.142
Once or several times a week vs. daily	−0.44	2.00	[−4.38; 3.49]	–	0.824
Once or several times a month vs. daily	−2.59	2.32	[−7.15; 1.97]	–	0.264
Once a year or less often vs. daily	−5.06	2.56	[−10.09; −0.03]	–	0.049
Perceived quality of family contact–overall predictor test	–	–	–	12.66 (2, 379)	<0.001
Partly good vs. good	7.38	2.02	[3.40; 11.36]	–	<0.001
Poor vs. good	19.66	5.15	[9.53; 29.79]	–	<0.001
Membership in a community organization–overall predictor test	–	–	–	1.13 (1, 379)	0.289
Member vs. non-member	−1.54	1.45	[−4.39; 1.31]	–	0.289

Note: Reference categories were male sex, urban area, married participants, living with a spouse or partner, daily family contact, good-quality family contact, and non-membership in a community organization. B—unstandardized regression coefficient; SEHC3—HC3 robust standard error; CI—confidence interval; FHC3—robust Wald F-statistic; df—degrees of freedom.

**Table 8:** Multiple linear regression model of family loneliness.

Predictor	B	SEHC3	95% CI	FHC3 (df)	*p*
Age	0.06	0.08	[−0.09; 0.22]	0.68 (1, 383)	0.411
Marital status–overall predictor test	–	–	–	1.45 (4, 383)	0.216
Cohabiting vs. married	0.09	1.81	[−3.46; 3.65]	–	0.959
Never married vs. married	−6.82	2.88	[−12.47; −1.16]	–	0.018
Divorced vs. married	−0.71	2.17	[−4.98; 3.56]	–	0.743
Widowed vs. married	−0.95	1.60	[−4.09; 2.19]	–	0.552
Household composition–overall predictor test	–	–	–	0.84 (2, 383)	0.430
Living alone vs. living with a spouse/partner	2.19	1.81	[−1.36; 5.75]	–	0.226
Living with other household members vs. living with a spouse/partner	0.35	1.26	[−2.12; 2.82]	–	0.780
Frequency of family contact–overall predictor test	–	–	–	1.32 (3, 383)	0.266
Once or several times a week vs. daily	−0.73	1.14	[−2.97; 1.51]	–	0.522
Once or several times a month vs. daily	0.82	1.51	[−2.16; 3.79]	–	0.590
Once a year or less often vs. daily	2.30	1.67	[−0.98; 5.58]	–	0.169
Perceived quality of family contact–overall predictor test	–	–	–	44.18 (2, 383)	<0.001
Partly good vs. good	7.17	1.40	[4.41; 9.92]	–	<0.001
Poor vs. good	25.52	3.01	[19.60; 31.44]	–	<0.001

Note: Reference categories were married participants, living with a spouse or partner, daily family contact, and good-quality family contact. B–unstandardized regression coefficient; SEHC3—HC3 robust standard error; CI—confidence interval; FHC3—robust Wald F-statistic; df—degrees of freedom.

**Table 9:** Multiple linear regression model of loneliness in romantic relationships.

Predictor	B	SEHC3	95% CI	FHC3 (df)	*p*
Age	0.24	0.08	[0.07; 0.40]	7.94 (1, 413)	0.005
Marital status–overall predictor test	–	–	–	53.50 (4, 413)	<0.001
Cohabiting vs. married	−3.95	4.33	[−12.46; 4.56]	–	0.362
Never married vs. married	14.66	3.85	[7.09; 22.22]	–	<0.001
Divorced vs. married	21.55	2.57	[16.49; 26.61]	–	<0.001
Widowed vs. married	15.58	1.19	[13.25; 17.91]	–	<0.001

Note: Married participants constituted the reference category. B—unstandardized regression coefficient; SEHC3—HC3 robust standard error; CI—confidence interval; FHC3—robust Wald F-statistic; df—degrees of freedom.

## Data Availability

The original data presented in the study are openly available on FigShare at https://doi.org/10.6084/m9.figshare.33154934.v1 (accessed on 4 August 2026).

## Supplementary Materials

The following supporting information can be downloaded at: https://www.mdpi.com/article/10.3390/healthcare14193165/s1, Table S1: Missing data and model-specific analytical sample sizes; Table S2: Regression sample-size sensitivity analysis; Table S3: Sensitivity analyses using the same measured covariate set for all three loneliness outcomes; Table S4: Sensitivity analyses after excluding the small cohabiting and never-married marital-status categories; Table S5: Sensitivity analyses after excluding participants who rated the perceived quality of family contact as poor; Table S6: Sensitivity analyses after combining partly good and poor family-contact quality into a single not-fully-good category; Table S7: Sensitivity analyses of loneliness in romantic relationships without marital status; Table S8: Multicollinearity and regression diagnostic checks for the primary models; Table S9: Influence diagnostics and sensitivity analyses after excluding observations with Cook’s distance > 4/N.

## Abbreviations

The following abbreviations are used in this manuscript:

CI	Confidence interval
df	Degrees of freedom
HC3	Heteroscedasticity-consistent covariance matrix estimator, type 3
M	Mean
Mdn	Median
Q1	First quartile
Q3	Third quartile
SD	Standard deviation
SE	Standard error
UCLA	University of California, Los Angeles
WHO	World Health Organization
