# Supplementary material for: Dimensions of Loneliness Among Older Adults in Osijek-Baranja County: Associations with Sociodemographic, Family, and Social Characteristics
Source: Healthcare (Basel). 2026 Sep 24;14(19):3165. doi: 10.3390/healthcare14193165 (PMC13665258; doi:10.3390/healthcare14193165)
Supplement: Supplementary file 1 [file healthcare-14-03165-s001.zip › healthcare-4527983-supplementary.pdf]

Supplementary Materials: Dimensions of Loneliness Among Older Adults in Osijek-Baranja County: Associations with Sociodemographic, Family, and Social Characteristics

Supplementary Methods

The primary regression models were retained as specified in the manuscript. Additional analyses were conducted as robustness checks. First, social, family, and romantic loneliness were re-estimated using an identical set of measured covariates: age, sex, place of residence, marital status, household composition, frequency and perceived quality of family contact, and membership in a community organization. Second, models involving marital status were repeated after excluding the small cohabiting (n = 8) and never-married (n = 12) categories. Third, because only 16 participants in the overall sample rated family-contact quality as poor, the social and family loneliness models were repeated after excluding this category and after combining partly good and poor contact into a single not-fully-good category. Fourth, loneliness in romantic relationships was re-estimated without marital status to assess the extent to which the model depended on this structurally related characteristic. Finally, influential observations were evaluated and the primary models were refitted after excluding observations with Cook's distance greater than 4/N. HC3 robust standard errors and robust Wald F-tests were used throughout. Missing data were not imputed; regression analyses used complete cases for variables included in each model. Interpretation of the sensitivity analyses emphasized the direction and magnitude of key coefficient estimates and their 95% confidence intervals rather than omnibus model significance alone. Because the cohabiting (n = 8), never-married (n = 12), and poor family-contact quality (n = 16) categories were small, primary-model estimates involving these groups were regarded as exploratory.

Table S1. Missing data and model-specific analytical sample sizes.

Panel A. Missing data by variable

Variable	Valid n	Missing n (%)
Age	431	0 (0.0%)
Sex	431	0 (0.0%)
Place of residence	431	0 (0.0%)
Marital status	421	10 (2.3%)
Household composition	423	8 (1.9%)
Frequency of family contact	414	17 (3.9%)
Perceived quality of family contact	424	7 (1.6%)
Membership in a community organization	431	0 (0.0%)
Social loneliness	431	0 (0.0%)
Family loneliness	431	0 (0.0%)
Loneliness in romantic relationships	429	2 (0.5%)

Panel B. Complete-case analytical sample sizes for the primary regression models

Outcome	Overall sample N	Analytical N	Excluded from model
Social loneliness	431	396	35
Family loneliness	431	396	35
Loneliness in romantic relationships	431	419	12

Note: n = number of participants; N = sample size. Percentages are based on the overall analytical sample of 431 participants. Regression models used complete cases for all variables included in the respective model.

Table S2. Regression sample-size sensitivity analysis.

Outcome	N	Predictor parameters	Residual df	Power	Minimum detectable f^2	Equivalent R^2
Social loneliness	396	16	379	0.80	0.050	0.048
Social loneliness	396	16	379	0.90	0.063	0.059
Family loneliness	396	12	383	0.80	0.045	0.043
Family loneliness	396	12	383	0.90	0.057	0.054
Loneliness in romantic relationships	419	5	413	0.80	0.031	0.030
Loneliness in romantic relationships	419	5	413	0.90	0.040	0.038

Note: N = analytical sample size; df = degrees of freedom; f^2 = Cohen's effect-size measure for multiple regression; R^2 = coefficient of determination. Calculations used $\alpha = 0.05$. The 80% power result for the most parameter-rich primary model (social loneliness; N = 396; 16 predictor parameters) was $f^2 = 0.050$.

Table S3. Sensitivity analyses using the same measured covariate set for all three loneliness outcomes.

Panel A. Social loneliness

Model fit: N = 396; FHC3(16, 379) = 4.49; $p < 0.001$; adjusted $R^2 = 0.130$.

Predictor or contrast	B	SEHC3	95% CI	FHC3 (df)	p
Age	0.37	0.15	[0.07; 0.66]	6.05 (1, 379)	0.014
Sex - overall predictor test	-	-	-	1.73 (1, 379)	0.190
Female vs. male	-2.03	1.54	[-5.07; 1.01]	-	0.190
Place of residence - overall predictor test	-	-	-	0.33 (2, 379)	0.719
Suburban vs. urban area	1.62	2.13	[-2.57; 5.81]	-	0.447
Rural vs. urban area	-0.16	1.85	[-3.79; 3.47]	-	0.931
Marital status - overall predictor test	-	-	-	5.73 (4, 379)	< 0.001
Cohabiting vs. married	-9.83	2.91	[-15.56; -4.10]	-	< 0.001
Never married vs. married	-9.63	3.64	[-16.78; -2.48]	-	0.008
Divorced vs. married	3.00	4.06	[-4.99; 10.99]	-	0.461
Widowed vs. married	-0.03	2.51	[-4.97; 4.91]	-	0.990
Household composition - overall predictor test	-	-	-	0.06 (2, 379)	0.944
Living alone vs. living with a spouse/partner	-0.96	2.90	[-6.67; 4.75]	-	0.741
Living with other household members vs. living with a spouse/partner	-0.52	2.16	[-4.76; 3.72]	-	0.809
Frequency of family contact - overall predictor test	-	-	-	1.83 (3, 379)	0.142
Once or several times a week vs. daily	-0.44	2.00	[-4.38; 3.49]	-	0.824
Once or several times a month vs. daily	-2.59	2.32	[-7.15; 1.97]	-	0.264
Once a year or less often vs. daily	-5.06	2.56	[-10.09; -0.03]	-	0.049
Perceived quality of family contact - overall predictor test	-	-	-	12.66 (2, 379)	< 0.001
Partly good vs. good	7.38	2.02	[3.40; 11.36]	-	< 0.001
Poor vs. good	19.66	5.15	[9.53; 29.79]	-	< 0.001
Membership in a community organization - overall predictor test	-	-	-	1.13 (1, 379)	0.289
Member vs. non-member	-1.54	1.45	[-4.39; 1.31]	-	0.289

Panel B. Family loneliness

Model fit: N = 396; FHC3(16, 379) = 7.61; $p < 0.001$; adjusted $R^2 = 0.275$.

Predictor or contrast	B	SEHC3	95% CI	FHC3 (df)	p
Age	0.05	0.08	[-0.10; 0.20]	0.50 (1, 379)	0.479
Sex - overall predictor test	-	-	-	0.04 (1, 379)	0.845
Female vs. male	-0.20	1.02	[-2.21; 1.81]	-	0.845
Place of residence - overall predictor test	-	-	-	2.45 (2, 379)	0.088
Suburban vs. urban area	3.21	1.63	[-0.01; 6.42]	-	0.050
Rural vs. urban area	-0.58	1.15	[-2.85; 1.68]	-	0.613
Marital status - overall predictor test	-	-	-	1.09 (4, 379)	0.359
Cohabiting vs. married	-0.31	1.77	[-3.79; 3.16]	-	0.859
Never married vs. married	-6.26	3.01	[-12.17; -0.34]	-	0.038
Divorced vs. married	-0.50	2.20	[-4.82; 3.82]	-	0.819
Widowed vs. married	-0.60	1.67	[-3.88; 2.69]	-	0.721
Household composition - overall predictor test	-	-	-	0.76 (2, 379)	0.469
Living alone vs. living with a spouse/partner	1.91	1.80	[-1.63; 5.45]	-	0.290
Living with other household members vs. living with a spouse/partner	-0.02	1.29	[-2.56; 2.51]	-	0.985
Frequency of family contact - overall predictor test	-	-	-	1.66 (3, 379)	0.176
Once or several times a week vs. daily	-0.60	1.19	[-2.95; 1.75]	-	0.615
Once or several times a month vs. daily	1.20	1.65	[-2.05; 4.44]	-	0.468
Once a year or less often vs. daily	2.85	1.72	[-0.54; 6.23]	-	0.099
Perceived quality of family contact - overall predictor test	-	-	-	32.85 (2, 379)	< 0.001
Partly good vs. good	7.05	1.40	[4.30; 9.81]	-	< 0.001
Poor vs. good	23.97	3.46	[17.16; 30.77]	-	< 0.001
Membership in a community organization - overall predictor test	-	-	-	0.00 (1, 379)	0.987
Member vs. non-member	0.02	1.04	[-2.03; 2.07]	-	0.987

Panel C. Loneliness in romantic relationships

Model fit: N = 394; FHC3(16, 377) = 24.05; $p < 0.001$; adjusted $R^2 = 0.438$.

Predictor or contrast	B	SEHC3	95% CI	FHC3 (df)	p
Age	0.30	0.09	[0.12; 0.47]	11.39 (1, 377)	< 0.001
Sex - overall predictor test	-	-	-	5.85 (1, 377)	0.016
Female vs. male	3.26	1.35	[0.61; 5.92]	-	0.016
Place of residence - overall predictor test	-	-	-	1.93 (2, 377)	0.146
Suburban vs. urban area	0.93	1.69	[-2.39; 4.24]	-	0.582
Rural vs. urban area	-2.08	1.40	[-4.82; 0.66]	-	0.137
Marital status - overall predictor test	-	-	-	10.06 (4, 377)	< 0.001
Cohabiting vs. married	-8.72	3.33	[-15.27; -2.18]	-	0.009
Never married vs. married	8.12	4.18	[-0.10; 16.34]	-	0.053
Divorced vs. married	16.21	3.57	[9.19; 23.23]	-	< 0.001
Widowed vs. married	9.88	2.22	[5.52; 14.23]	-	< 0.001
Household composition - overall predictor test	-	-	-	5.14 (2, 377)	0.006
Living alone vs. living with a spouse/partner	5.70	2.58	[0.63; 10.77]	-	0.028
Living with other household members vs. living with a spouse/partner	-0.17	1.91	[-3.93; 3.59]	-	0.927
Frequency of family contact - overall predictor test	-	-	-	1.59 (3, 377)	0.191
Once or several times a week vs. daily	-1.29	1.55	[-4.34; 1.77]	-	0.408
Once or several times a month vs. daily	-1.75	1.94	[-5.56; 2.07]	-	0.368
Once a year or less often vs. daily	2.03	2.00	[-1.90; 5.95]	-	0.311
Perceived quality of family contact - overall predictor test	-	-	-	10.75 (2, 377)	< 0.001
Partly good vs. good	3.37	1.50	[0.42; 6.32]	-	0.025
Poor vs. good	10.36	2.33	[5.78; 14.95]	-	< 0.001
Membership in a community organization - overall predictor test	-	-	-	0.84 (1, 377)	0.359
Member vs. non-member	1.22	1.33	[-1.39; 3.84]	-	0.359

Note: B = unstandardized regression coefficient; SEHC3 = HC3 robust standard error; CI = confidence interval; FHC3 = robust Wald F-statistic; df = degrees of freedom; R^2 = coefficient of determination. Reference categories were male sex, urban area, married participants, living with a spouse/partner, daily family contact, good-quality family contact, and non-membership in a community organization. The same measured covariate set was included in all three models.

Table S4. Sensitivity analyses after excluding the small cohabiting and never-married marital-status categories.

Panel A. Social loneliness

Model fit: N = 376; FHC3(14, 361) = 3.36; $p < 0.001$; adjusted $R^2 = 0.110$.

Predictor or contrast	B	SEHC3	95% CI	FHC3 (df)	p
Age	0.39	0.16	[0.08; 0.70]	5.99 (1, 361)	0.015
Sex - overall predictor test	-	-	-	1.24 (1, 361)	0.266
Female vs. male	-1.83	1.64	[-5.06; 1.40]	-	0.266
Place of residence - overall predictor test	-	-	-	0.28 (2, 361)	0.757
Suburban vs. urban area	1.41	2.22	[-2.96; 5.77]	-	0.526
Rural vs. urban area	-0.45	1.92	[-4.22; 3.32]	-	0.816
Marital status - overall predictor test	-	-	-	0.41 (2, 361)	0.663
Divorced vs. married	3.03	4.23	[-5.29; 11.35]	-	0.474
Widowed vs. married	-0.15	2.62	[-5.31; 5.01]	-	0.954
Household composition - overall predictor test	-	-	-	0.06 (2, 361)	0.941
Living alone vs. living with a spouse/partner	-1.04	3.09	[-7.12; 5.04]	-	0.736
Living with other household members vs. living with a spouse/partner	-0.22	2.22	[-4.59; 4.16]	-	0.922
Frequency of family contact - overall predictor test	-	-	-	1.49 (3, 361)	0.218
Once or several times a week vs. daily	-0.54	2.04	[-4.56; 3.48]	-	0.791
Once or several times a month vs. daily	-2.33	2.41	[-7.06; 2.41]	-	0.334
Once a year or less often vs. daily	-4.98	2.68	[-10.25; 0.29]	-	0.064
Perceived quality of family contact - overall predictor test	-	-	-	9.74 (2, 361)	< 0.001
Partly good vs. good	7.43	2.13	[3.24; 11.62]	-	< 0.001
Poor vs. good	19.29	6.60	[6.31; 32.26]	-	0.004
Membership in a community organization - overall predictor test	-	-	-	0.88 (1, 361)	0.348
Member vs. non-member	-1.40	1.49	[-4.33; 1.53]	-	0.348

Panel B. Family loneliness

Model fit: N = 376; FHC3(10, 365) = 9.03; $p < 0.001$; adjusted $R^2 = 0.247$.

Predictor or contrast	B	SEHC3	95% CI	FHC3 (df)	p
Age	0.06	0.08	[-0.10; 0.22]	0.59 (1, 365)	0.442
Marital status - overall predictor test	-	-	-	0.07 (2, 365)	0.931
Divorced vs. married	-0.48	2.26	[-4.93; 3.97]	-	0.832
Widowed vs. married	-0.63	1.67	[-3.91; 2.66]	-	0.708
Household composition - overall predictor test	-	-	-	0.46 (2, 365)	0.631
Living alone vs. living with a spouse/partner	1.69	1.91	[-2.07; 5.44]	-	0.378
Living with other household members vs. living with a spouse/partner	0.23	1.30	[-2.33; 2.80]	-	0.858
Frequency of family contact - overall predictor test	-	-	-	1.40 (3, 365)	0.243
Once or several times a week vs. daily	-0.79	1.15	[-3.06; 1.48]	-	0.492
Once or several times a month vs. daily	1.09	1.56	[-1.98; 4.17]	-	0.484
Once a year or less often vs. daily	2.35	1.76	[-1.11; 5.81]	-	0.182
Perceived quality of family contact - overall predictor test	-	-	-	34.48 (2, 365)	< 0.001
Partly good vs. good	7.53	1.47	[4.65; 10.41]	-	< 0.001
Poor vs. good	25.82	3.78	[18.38; 33.26]	-	< 0.001

Panel C. Loneliness in romantic relationships

Model fit: N = 399; FHC3(3, 395) = 86.84; $p < 0.001$; adjusted $R^2 = 0.388$.

Predictor or contrast	B	SEHC3	95% CI	FHC3 (df)	p
Age	0.23	0.08	[0.07; 0.40]	7.61 (1, 395)	0.006
Marital status - overall predictor test	-	-	-	101.64 (2, 395)	< 0.001
Divorced vs. married	21.54	2.57	[16.48; 26.60]	-	< 0.001
Widowed vs. married	15.60	1.19	[13.26; 17.93]	-	< 0.001

Note: B = unstandardized regression coefficient; SEHC3 = HC3 robust standard error; CI = confidence interval; FHC3 = robust Wald F-statistic; df = degrees of freedom; R^2 = coefficient of determination. Cohabiting participants (n = 8) and never-married participants (n = 12) were excluded. The marital-status reference category was married; the remaining contrasts were divorced and widowed versus married. Other reference categories were unchanged from the primary models.

This exclusion analysis was used to assess the stability of estimates for the remaining marital-status groups; estimates involving the very small excluded categories in the primary models should be considered exploratory.

Table S5. Sensitivity analyses after excluding participants who rated the perceived quality of family contact as poor.

Panel A. Social loneliness

Model fit: N = 381; FHC3(15, 365) = 4.01; $p < 0.001$; adjusted $R^2 = 0.084$.

Predictor or contrast	B	SEHC3	95% CI	FHC3 (df)	p
Age	0.37	0.15	[0.06; 0.67]	5.69 (1, 365)	0.018
Sex - overall predictor test	-	-	-	3.00 (1, 365)	0.084
Female vs. male	-2.70	1.56	[-5.76; 0.37]	-	0.084
Place of residence - overall predictor test	-	-	-	0.60 (2, 365)	0.551
Suburban vs. urban area	2.22	2.13	[-1.96; 6.40]	-	0.297
Rural vs. urban area	-0.14	1.87	[-3.81; 3.53]	-	0.941
Marital status - overall predictor test	-	-	-	5.58 (4, 365)	< 0.001
Cohabiting vs. married	-10.36	3.15	[-16.56; -4.15]	-	0.001
Never married vs. married	-10.28	4.05	[-18.25; -2.31]	-	0.012
Divorced vs. married	3.74	4.29	[-4.69; 12.17]	-	0.384
Widowed vs. married	-0.70	2.56	[-5.72; 4.33]	-	0.786
Household composition - overall predictor test	-	-	-	0.02 (2, 365)	0.984
Living alone vs. living with a spouse/partner	-0.47	3.02	[-6.41; 5.47]	-	0.876
Living with other household members vs. living with a spouse/partner	-0.34	2.18	[-4.63; 3.94]	-	0.874
Frequency of family contact - overall predictor test	-	-	-	1.34 (3, 365)	0.261
Once or several times a week vs. daily	-0.37	2.00	[-4.31; 3.57]	-	0.854
Once or several times a month vs. daily	-2.15	2.37	[-6.81; 2.51]	-	0.364
Once a year or less often vs. daily	-4.35	2.60	[-9.46; 0.75]	-	0.094
Perceived quality of family contact - overall predictor test	-	-	-	12.72 (1, 365)	< 0.001
Partly good vs. good	7.22	2.02	[3.24; 11.20]	-	< 0.001
Membership in a community organization - overall predictor test	-	-	-	0.66 (1, 365)	0.417

Member vs. non-member	-1.19	1.47	[-4.09; 1.70]	-	0.417

Panel B. Family loneliness

Model fit: N = 381; FHC3(11, 369) = 3.96; $p < 0.001$; adjusted $R^2 = 0.100$.

Predictor or contrast	B	SEHC3	95% CI	FHC3 (df)	p
Age	0.07	0.08	[-0.08; 0.23]	0.83 (1, 369)	0.364
Marital status - overall predictor test	-	-	-	0.94 (4, 369)	0.438
Cohabiting vs. married	-0.41	1.95	[-4.25; 3.43]	-	0.835
Never married vs. married	-6.41	3.42	[-13.14; 0.31]	-	0.061
Divorced vs. married	-0.71	2.31	[-5.25; 3.84]	-	0.760
Widowed vs. married	-1.53	1.64	[-4.74; 1.69]	-	0.352
Household composition - overall predictor test	-	-	-	1.13 (2, 369)	0.324
Living alone vs. living with a spouse/partner	2.60	1.88	[-1.09; 6.29]	-	0.167
Living with other household members vs. living with a spouse/partner	0.35	1.27	[-2.15; 2.86]	-	0.782
Frequency of family contact - overall predictor test	-	-	-	1.56 (3, 369)	0.199
Once or several times a week vs. daily	-0.65	1.14	[-2.90; 1.59]	-	0.568
Once or several times a month vs. daily	1.56	1.52	[-1.44; 4.56]	-	0.307
Once a year or less often vs. daily	2.36	1.71	[-1.01; 5.72]	-	0.169
Perceived quality of family contact - overall predictor test	-	-	-	24.83 (1, 369)	< 0.001
Partly good vs. good	7.05	1.42	[4.27; 9.84]	-	< 0.001

Note: B = unstandardized regression coefficient; SEHC3 = HC3 robust standard error; CI = confidence interval; FHC3 = robust Wald F-statistic; df = degrees of freedom; R^2 = coefficient of determination. The overall study sample contained 16 participants who rated family-contact quality as poor; these participants were excluded from this sensitivity analysis. Good-quality contact remained the reference category, with partly good contact as the remaining contrast. Estimates involving the poor-contact group in the primary models should be considered exploratory because of the small group size.

Table S6. Sensitivity analyses after combining partly good and poor family-contact quality into a single not-fully-good category.

Panel A. Social loneliness

Model fit: N = 396; FHC3(15, 380) = 4.58; $p < 0.001$; adjusted $R^2 = 0.111$.

Predictor or contrast	B	SEHC3	95% CI	FHC3 (df)	p
Age	0.34	0.15	[0.05; 0.63]	5.25 (1, 380)	0.022
Sex - overall predictor test	-	-	-	2.68 (1, 380)	0.102
Female vs. male	-2.47	1.51	[-5.44; 0.50]	-	0.102
Place of residence - overall predictor test	-	-	-	1.08 (2, 380)	0.341
Suburban vs. urban area	2.95	2.08	[-1.15; 7.05]	-	0.158
Rural vs. urban area	-0.09	1.85	[-3.73; 3.56]	-	0.963
Marital status - overall predictor test	-	-	-	3.98 (4, 380)	0.004
Cohabiting vs. married	-8.45	3.17	[-14.67; -2.22]	-	0.008
Never married vs. married	-7.50	3.95	[-15.28; 0.27]	-	0.058
Divorced vs. married	3.77	4.01	[-4.12; 11.66]	-	0.348
Widowed vs. married	0.69	2.59	[-4.40; 5.78]	-	0.790
Household composition - overall predictor test	-	-	-	0.07 (2, 380)	0.932
Living alone vs. living with a spouse/partner	-1.00	2.97	[-6.85; 4.84]	-	0.736
Living with other household members vs. living with a spouse/partner	-0.71	2.19	[-5.02; 3.61]	-	0.748
Frequency of family contact - overall predictor test	-	-	-	1.67 (3, 380)	0.173
Once or several times a week vs. daily	-0.95	2.02	[-4.92; 3.02]	-	0.637
Once or several times a month vs. daily	-2.77	2.35	[-7.39; 1.84]	-	0.238
Once a year or less often vs. daily	-5.11	2.56	[-10.14; -0.08]	-	0.047
Perceived quality of family contact - overall predictor test	-	-	-	21.17 (1, 380)	< 0.001
Not fully good vs. good	9.31	2.02	[5.33; 13.28]	-	< 0.001
Membership in a community organization - overall predictor test	-	-	-	1.78 (1, 380)	0.183
Member vs. non-member	-1.94	1.45	[-4.79; 0.92]	-	0.183

Panel B. Family loneliness

Model fit: N = 396; FHC3(11, 384) = 6.46; $p < 0.001$; adjusted $R^2 = 0.177$.

Predictor or contrast	B	SEHC3	95% CI	FHC3 (df)	p
Age	0.04	0.08	[-0.11; 0.20]	0.27 (1, 384)	0.606
Marital status - overall predictor test	-	-	-	0.41 (4, 384)	0.802
Cohabiting vs. married	2.06	2.96	[-3.75; 7.87]	-	0.486
Never married vs. married	-3.78	3.65	[-10.96; 3.41]	-	0.302
Divorced vs. married	0.21	2.39	[-4.49; 4.90]	-	0.931
Widowed vs. married	-0.50	1.83	[-4.10; 3.10]	-	0.785
Household composition - overall predictor test	-	-	-	0.81 (2, 384)	0.447
Living alone vs. living with a spouse/partner	2.44	2.04	[-1.57; 6.46]	-	0.231
Living with other household members vs. living with a spouse/partner	0.43	1.32	[-2.16; 3.03]	-	0.744
Frequency of family contact - overall predictor test	-	-	-	1.75 (3, 384)	0.155
Once or several times a week vs. daily	-1.63	1.20	[-4.00; 0.73]	-	0.176
Once or several times a month vs. daily	0.38	1.60	[-2.76; 3.52]	-	0.811
Once a year or less often vs. daily	1.68	1.79	[-1.83; 5.19]	-	0.347
Perceived quality of family contact - overall predictor test	-	-	-	43.77 (1, 384)	< 0.001
Not fully good vs. good	10.27	1.55	[7.21; 13.32]	-	< 0.001

Note: B = unstandardized regression coefficient; SEHC3 = HC3 robust standard error; CI = confidence interval; FHC3 = robust Wald F-statistic; df = degrees of freedom; R^2 = coefficient of determination. Perceived family-contact quality was dichotomized as good versus not fully good (partly good + poor); good-quality contact was the reference category. Other reference categories were unchanged from the primary models.

Table S7. Sensitivity analyses of loneliness in romantic relationships without marital status.

Panel A. Model-level comparison

Model	N	Predictor parameters	F_{HC3} (df)	p	Adjusted R^2
Primary model: age + marital status	419	5	54.73 (5, 413)	< 0.001	0.385
Sensitivity: age only	429	1	18.89 (1, 427)	< 0.001	0.035
Sensitivity: remaining measured covariates without marital status	402	12	22.06 (12, 389)	< 0.001	0.358

Panel B. Age-only model

Model fit: N = 429; $F_{HC3}(1, 427) = 18.89$; $p < 0.001$; adjusted $R^2 = 0.035$.

Predictor or contrast	B	SE_{HC3}	95% CI	F_{HC3} (df)	p
Age	0.43	0.10	[0.23; 0.62]	18.89 (1, 427)	< 0.001

Panel C. Model including the remaining measured covariates without marital status

Model fit: N = 402; $F_{HC3}(12, 389) = 22.06$; $p < 0.001$; adjusted $R^2 = 0.358$.

Predictor or contrast	B	SE_{HC3}	95% CI	F_{HC3} (df)	p
Age	0.27	0.09	[0.08; 0.45]	8.21 (1, 389)	0.004
Sex - overall predictor test	-	-	-	7.71 (1, 389)	0.006
Female vs. male	3.75	1.35	[1.09; 6.41]	-	0.006
Place of residence - overall predictor test	-	-	-	1.56 (2, 389)	0.211
Suburban vs. urban area	0.05	1.78	[-3.44; 3.54]	-	0.979
Rural vs. urban area	-2.36	1.43	[-5.18; 0.46]	-	0.101
Household composition - overall predictor test	-	-	-	66.26 (2, 389)	< 0.001
Living alone vs. living with a spouse/partner	16.35	1.48	[13.45; 19.26]	-	< 0.001
Living with other household members vs. living with a spouse/partner	4.79	1.72	[1.41; 8.17]	-	0.006
Frequency of family contact - overall predictor test	-	-	-	1.08 (3, 389)	0.356

Once or several times a week vs. daily	-1.34	1.65	[-4.58; 1.90]	-	0.416
Once or several times a month vs. daily	-1.04	2.05	[-5.07; 3.00]	-	0.614
Once a year or less often vs. daily	1.70	2.13	[-2.49; 5.89]	-	0.425
Perceived quality of family contact - overall predictor test	-	-	-	10.47 (2, 389)	< 0.001
Partly good vs. good	4.08	1.51	[1.12; 7.04]	-	0.007
Poor vs. good	11.33	2.84	[5.74; 16.91]	-	< 0.001
Membership in a community organization - overall predictor test	-	-	-	0.93 (1, 389)	0.336
Member vs. non-member	1.30	1.35	[-1.35; 3.95]	-	0.336

Note: B = unstandardized regression coefficient; SEHC3 = HC3 robust standard error; CI = confidence interval; FHC3 = robust Wald F-statistic; df = degrees of freedom; R^2 = coefficient of determination. In Panel C, reference categories were male sex, urban area, living with a spouse/partner, daily family contact, good-quality family contact, and non-membership in a community organization. These analyses were performed to assess the extent to which the romantic-loneliness findings depended on inclusion of marital status.

Table S8. Multicollinearity and regression diagnostic checks for the primary models.

Panel A. Multicollinearity diagnostics

Model	Predictor	GVIF	df	Adjusted GVIF
Social loneliness	Age	1.302	1	1.141
Social loneliness	Sex	1.207	1	1.098
Social loneliness	Place of residence	1.333	2	1.075
Social loneliness	Marital status	4.179	4	1.196
Social loneliness	Household composition	4.069	2	1.420
Social loneliness	Frequency of family contact	1.785	3	1.101
Social loneliness	Perceived quality of family contact	1.238	2	1.055
Social loneliness	Membership in a community organization	1.212	1	1.101
Family loneliness	Age	1.191	1	1.091
Family loneliness	Marital status	3.513	4	1.170
Family loneliness	Household composition	3.848	2	1.401
Family loneliness	Frequency of family contact	1.595	3	1.081
Family loneliness	Perceived quality of family contact	1.143	2	1.034
Loneliness in romantic relationships	Age	1.169	1	1.081
Loneliness in romantic relationships	Marital status	1.169	4	1.020

Panel B. Regression diagnostic tests

Model	Diagnostic check	Statistic	p
Social loneliness	Shapiro-Wilk test of residuals	0.981	< 0.001
Social loneliness	Breusch-Pagan test	32.501	0.009
Social loneliness	Ramsey RESET test	0.131	0.877
Social loneliness	Quadratic age term	0.363	0.547
Family loneliness	Shapiro-Wilk test of residuals	0.932	< 0.001
Family loneliness	Breusch-Pagan test	9.170	0.688
Family loneliness	Ramsey RESET test	3.509	0.031
Family loneliness	Quadratic age term	1.415	0.235
Loneliness in romantic relationships	Shapiro-Wilk test of residuals	0.995	0.253

Loneliness in romantic relationships	Breusch-Pagan test	14.920	0.011
Loneliness in romantic relationships	Ramsey RESET test	0.521	0.594
Loneliness in romantic relationships	Quadratic age term	1.817	0.178

Note: GVIF = generalized variance inflation factor; df = degrees of freedom; adjusted GVIF = GVIF^(1/(2×df)). The diagnostic statistic is W for the Shapiro-Wilk test, the Breusch-Pagan test statistic for heteroscedasticity, F for the Ramsey RESET test, and the HC3 robust Wald F-statistic for the quadratic age term. HC3 robust standard errors were used in all reported regression models because heteroscedasticity was detected in the social and romantic-loneliness models.

Table S9. Influence diagnostics and sensitivity analyses after excluding observations with Cook's distance > 4/N.

Panel A. Number of observations exceeding influence-diagnostic thresholds

Model	N	Cook > 4/N	High leverage	|Studentized residual| > 3	High DFFITS	High DFBETAS
Family loneliness	396	23	36	2	23	123
Loneliness in romantic relationships	419	21	55	2	21	62
Social loneliness	396	23	32	2	23	128

Panel B. Model-level sensitivity after excluding observations with Cook's distance > 4/N

Outcome	N after exclusion	Excluded n	FHC3 (df)	p	Adjusted R^2
Social loneliness	373	23	7.32 (16, 356)	< 0.001	0.157
Family loneliness	373	23	16.25 (12, 360)	< 0.001	0.269
Loneliness in romantic relationships	398	21	75.01 (5, 392)	< 0.001	0.431

Panel C. Overall predictor tests after exclusion based on Cook's distance

Outcome	Predictor	FHC3 (df)	p
Social loneliness	Age	23.53 (1, 356)	< 0.001
Social loneliness	Sex	1.16 (1, 356)	0.283
Social loneliness	Place of residence	4.35 (2, 356)	0.014
Social loneliness	Marital status	3.47 (4, 356)	0.008
Social loneliness	Household composition	0.61 (2, 356)	0.545
Social loneliness	Frequency of family contact	3.21 (3, 356)	0.023
Social loneliness	Perceived quality of family contact	15.81 (2, 356)	< 0.001
Social loneliness	Membership in a community organization	1.79 (1, 356)	0.182
Family loneliness	Age	0.17 (1, 360)	0.684
Family loneliness	Marital status	2.84 (4, 360)	0.024
Family loneliness	Household composition	3.34 (2, 360)	0.036
Family loneliness	Frequency of family contact	1.07 (3, 360)	0.361
Family loneliness	Perceived quality of family contact	74.58 (2, 360)	< 0.001
Loneliness in romantic relationships	Age	7.92 (1, 392)	0.005

Loneliness in romantic relationships	Marital status	89.49 (4, 392)	< 0.001

Note: N = analytical sample size; n = number of excluded observations; FHC3 = robust Wald F-statistic; df = degrees of freedom; R^2 = coefficient of determination; DFFITS = difference in fits; DFBETAS = standardized change in a regression coefficient after deleting an observation. The Cook's-distance sensitivity analysis excluded 23 observations from the social-loneliness model, 23 from the family-loneliness model, and 21 from the romantic-loneliness model. These analyses were used as robustness checks and did not replace the primary models.
